# The potential mechanisms and application prospects of non-coding RNAs in intervertebral disc degeneration

**DOI:** 10.3389/fendo.2022.1081185

**Published:** 2022-12-08

**Authors:** Chao Jiang, Zhe Chen, Xiaohui Wang, Yongyuan Zhang, Xinyu Guo, Zhengwei Xu, Hao Yang, Dingjun Hao

**Affiliations:** ^1^ Department of Spine Surgery, Hong Hui Hospital, Xi’an Jiaotong University, Xi’an, China; ^2^ Department of Developmental Genetics, Max Planck Institute for Heart and Lung Research, Bad Nauheim, Germany; ^3^ Translational Medicine Center, Hong Hui Hospital, Xi’an Jiaotong University, Xi’an, China

**Keywords:** non-coding RNAs, microRNAs, long noncoding RNAs, circular RNAs, intervertebral disc degeneration

## Abstract

Low back pain (LBP) is one of the most common musculoskeletal symptoms and severely affects patient quality of life. The majority of people may suffer from LBP during their life-span, which leading to huge economic burdens to family and society. According to the series of the previous studies, intervertebral disc degeneration (IDD) is considered as the major contributor resulting in LBP. Furthermore, non-coding RNAs (ncRNAs), mainly including microRNAs (miRNAs), long noncoding RNAs (lncRNAs) and circular RNAs (circRNAs), can regulate diverse cellular processes, which have been found to play pivotal roles in the development of IDD. However, the potential mechanisms of action for ncRNAs in the processes of IDD are still completely unrevealed. Therefore, it is challenging to consider ncRNAs to be used as the potential therapeutic targets for IDD. In this paper, we reviewed the current research progress and findings on ncRNAs in IDD: i). ncRNAs mainly participate in the process of IDD through regulating apoptosis of nucleus pulposus (NP) cells, metabolism of extracellular matrix (ECM) and inflammatory response; ii). the roles of miRNAs/lncRNAs/circRNAs are cross-talk in IDD development, which is similar to the network and can modulate each other; iii). ncRNAs have been attempted to combat the degenerative processes and may be promising as an efficient bio-therapeutic strategy in the future. Hence, this review systematically summarizes the principal pathomechanisms of IDD and shed light on the therapeutic potentials of ncRNAs in IDD.

## Introduction

Currently, low back pain (LBP) serves as one of the most prevalent musculoskeletal symptoms and has severe effects on the quality of life of individuals in the worldwide (1–3). Previous studies indicated that almost all people may have suffered from LBP during their life-span, resulting in substantial distress and economic burden (3, 4). With regard to the causes for LBP, it is still not completely unraveled, but intervertebral disc degeneration (IDD) is considered as the major contributor (5). IDD is a series of physiological and pathological changes occurring in the aging and degeneration of the intervertebral disc (IVD). A cross-sectional study indicated that almost 40% individuals suffered from IDD are less than 30 years, which is as high as 90% between 50 and 55 years (6). Nonetheless, there is lack of effective bio-therapeutic strategies for IDD and surgical intervention is hard to avoid in the final stage (7). Therefore, it is greatly necessary to clarify the underlying mechanisms of IDD at a cellular and molecular level.

**Figure 1:**
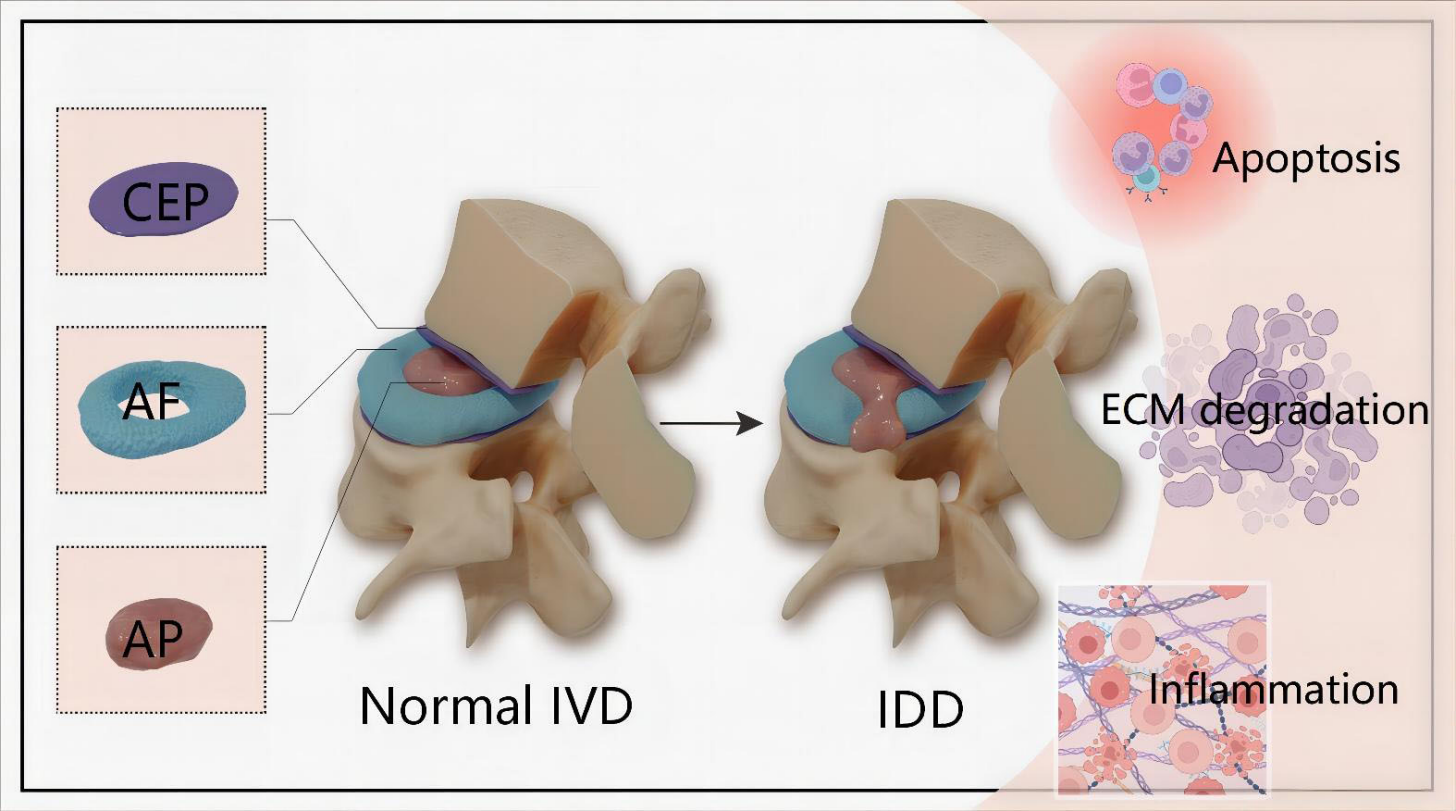
Schematic illustration of the anatomical structure of normal IVD and main pathophysiological features of IDD. IVD is composed of the central NP tissue, AF surrounding the NP, and CEP adhering the upper and lower vertebrae.The pathophysiological features of IDD are mainly characterized by cells apoptosis, imbalance of ECM and inflammatory response. IVD, intervertebral disc; IDD, intervertebral disc degeneration; CEP, cartilaginous endplate; AF, annulus fibrosus; NP, nucleus pulposus; ECM, extracellular matrix.

IDD is a long and chronic process accompanying structural failure and progressive aging of the normal intervertebral disc (IVD), which is attributed to series of factors including lifestyle, aging, genetic predispositions and excessively mechanical loading (8, 9). IVD is the fibrocartilage tissue structure between each two vertebrae and serve as crucial role in maintaining the stability of the spine. In terms of anatomical structure, IVD is composed of nucleus pulposus (NP) cells, annulus fibrosus (AF) and cartilaginous endplate (CEP) (**Figure 1**). Accumulating evidences demonstrate that genetic and environmental factors contribute to IDD, but the exact molecular mechanisms are still largely unclear (10). The pathophysiological processes of IDD are mainly characterized by cells apoptosis, imbalance of extracellular matrix (ECM) and inflammatory response (**Figure 1**).

Noncoding RNAs (ncRNAs), primarily including microRNAs (miRNAs), long ncRNAs (lncRNAs) and circular RNAs (circRNAs), have been found to exert extensive effects on biological processes such as cell proliferation, apoptosis and ECM metabolism (4, 11, 12). Furthermore, compelling evidences supported that the expression of ncRNAs are significantly different between IDD and control samples, implicating ncRNAs play crucial roles in the development of IDD (13–15). Notably, novel ncRNAs have been constantly identified through microarray, RNA sequencing and reverse transcription-quantitative PCR (RT-qPCR) (16, 17), which has attracted a large number of interesting researcher’s attentions on the functions and specific molecular mechanisms of ncRNAs in IDD. There is no doubt that these works provide excellent value of understanding the precise roles of ncRNAs and display promising future for IDD bio-therapeutic strategy.

In this review, we systematically summarized the literature of past five years on ncRNAs in the pathophysiological processes of IDD, mainly involving miRNAs, lncRNAs and circRNAs. In addition, the application prospects of ncRNAs as bio-therapeutic strategy for effective treatment of IDD are also discussed.

## The roles of miRNAs in the development of IDD

MiRNAs, a group of endogenous ncRNAs with 20–24bp nucleotides in length, can regulate gene expression through recognizing and targeting the complementary 3′untranslated regions (3′UTRs) of particular mRNAs. Previous studies demonstrated that miRNAs were significantly differentially expressed between IDD and non-IDD, and acted as pivotal roles in the development of IDD (11, 18).

### MiRNAs regulate the apoptosis of cells

The apoptosis of NP cells is a typical feature of IDD and a myriad of studies reported that miRNAs mediated the NP cells apoptosis through regulating specific gene expression (18). Previously, Ji et al. firstly confirmed that miR-141 causes spontaneous progression of IDD by means of a study in miR-141 KO and wild-type mice (19). Mechanistically, miR-141 induced NP cell apoptosis and facilitated IDD progress *via* the regulation of downstream SIRT1/NF-κB axis (19). In addition, silencing of miR-141 had a protective effect on IDD mice, while upregulating miR-141 accelerated the development of IDD. Similar to miR-141, a large body of studies demonstrated that miRNAs were up-regulated in DD and aggravated NP cells apoptosis, such as miR-96/FRS2 (20), miR-4478/MTH1 (21) and miR-328-5p/WWP2 (22). On the contrary, there were substantial numbers of miRNAs were down-regulated and most likely exert anti-apoptotic effects in IDD, mainly involving miR-129-5p/BMP2 (23), miR-623/CXCL12 (24) and miR-155-3p/KDM3A/HIF1α (25). Therefore, miRNA-mediated apoptosis of NP cells affected the pathological process of IDD *via* downstream hub proteins or signal pathway. Notably, apart from anti-apoptosis, miR-623/CXCL12 axis inhibited senescence in LPS-induced NP cells (24), which suggested that miRNAs likely play an important role in IDD development by affecting cellular senescence.

Given the particular structure of IVD, CEP is essential for NP cells to acquire nutrition and prevent damaging factors into the disc. Previous studies showed that CEP cells apoptosis or calcification induced CEP degeneration, which resulted in nutrient loss and rendered IDD progress (26, 27). In a previous experiment, overexpression of miR-34a was evidenced to promote Fas-induced CEP cells apoptosis through inhibiting Bcl-2 (28). Additionally, miR-20a/ANKH-mediated stiff matrix enhanced calcification of CEP, whereas suppression of miR-20a alleviated the gene expression of calcification (29). Likewise, miR-221 positively regulated CEP cells apoptosis by targeting estrogen receptor α (ERα) (30). Based on the findings above, miRNAs have been shown to contribute to CEP cells apoptosis and calcification, which is closely pertinent to the development of IDD. However, recent literature demonstrated that miR-142-3p knockdown promoted apoptosis and autophagy of CEP cells by HMGB1 (31), implying that miR-142-3p is likely to have potential in protecting IDD. Apart from the foregoing mechanisms, miR-106a-5p also mediates AF cells apoptosis, which can be suppressed by melatonin (32).

Taken together, miRNAs-mediated cells apoptosis (NP, CEP and AF cells) regulates the onset of IDD, which provides potential targets for intervening IDD. Nevertheless, the roles of miRNAs in NP cells apoptosis attracted more attention. In the future, the related-studies should pay more attention to the effects of miRNAs on CEP and AF cells apoptosis.

### MiRNAs regulate the metabolism of ECM

ECM, a non-cellular three-dimensional macromolecular network predominantly composed of collagens and proteoglycan, is indispensable to maintain normal IVD cell function. With regard to the pathological process of IDD, the imbalance of ECM metabolism is a biological hallmark for IDD, which is characterized by synthesis decrease and degradation increase (9, 33). The metabolism of ECM is determined by proteolytic enzymes, such as matrix metalloproteinases (MMPs) and growth differentiation factor 5 (GDF5). miRNAs have been evidenced to play crucial roles in ECM degradation through modulating the activity of essential enzymes.

MMPs have acted as an essential factor for modulating metabolism of ECM. It has been reported that miR-127-5p was downregulated in degenerated NP tissues and inhibited the expression of type II collagen (34). Further experiment confirmed that miR-127-5p mediated the degradation of ECM components through enhancing MMP-13 expression. Another study found that the level of miR-210 remarkably increased in human degenerated NP cells, resulting in suppression of autophagy-related gene 7 (ATG7) and elevation of MMP-13 and MMP-3 (35). Notably, ATG7 knock-down seriously undermined the influences of miR-210 inhibitor on MMP-13 and MMP-3. These findings indicated that miR-210-induced ECM degradation was attributed to the proteolytic activity of upregulated MMP-13 and MMP-3 by directly targeting ATG7. Similarly, Wang et al. (36) also evidenced that miR-21 contributed to ECM catabolism by inhibiting autophagy through PTEN/Akt/mTOR signal pathway and elicited upregulation of MMP-9 and MMP-3 expression. Collectively, miRNAs play critical roles in MMPs-mediated metabolism of ECM *via* modulating autophagy.

GDF5, a crucial member of bone morphogenetic protein family, has been found to exert vital effects in musculoskeletal physiological process (37, 38). More importantly, recent publications also revealed that GDF5 provided protective effects against IDD through inhibiting ECM catabolism and promoting ECM anabolism (39, 40). Specifically, miR-132 contributed to the degradation of ECM by inhibiting GDF5, whereas antagomiR-132 protected ECM from degradation in IDD rats (39). Likewise, upregulation of miR-665 enhanced expression of catabolic genes (MMP13 and ADAMTS4) *via* specifically binding to GDF5, leading to ECM degradation (40). Consequently, GDF5 is likely to be an important target in ECM degradation caused by miRNAs.

It has been evidenced that SRY-related high mobility group box (SOX) is involved in the process of IDD (41). MiR-30d was found to be upregulated in degenerative NP tissues, and suppression of miR-30d resulted in hypoactive catabolism of ECM *in vitro* (42). Notably, bioinformatics analysis demonstrated that SOX9, an important transcription factor, was a direct target regulated by miR-30d. Interestingly, overexpression of miR-499a-5p is propitious to prevent NP cells apoptosis and ECM decomposition through repressing SOX4 expression (43). Although SOX9 and SOX4 belong to the transcription factors, they show opposite effects on ECM in the development of IDD. In addition, there are some miRNAs mediated ECM metabolism, such as miR-154/FGF14 (44), miR-145/ADAM17 (45) and miR-1260b/TCF7L2 (46) axis. This implicates a wide biofunction of miRNAs in the IDD process.

In summary, ECM metabolism mediated by miRNAs has affected the progress of IDD. Targeting miRNAs may be a promising approach for maintenance of the proper balance of ECM catabolism and anabolism, thereby delaying the pathological change of IDD.

### MiRNAs regulate the inflammation

Inflammation is widely envisioned as one of pathological features accompanying with IDD (47, 48). Up to now, accumulating evidence have also shown that multiple miRNAs are involved in regulating the inflammatory response of IDD (49–51).

Nuclear factor κB (NF-κB) is a key signal pathway in inflammatory response. The activation of NF-κB promotes the inflammatory cascade, leading to adverse environment for NP cells and impetus to degeneration of IVD (48). With regard to miRNAs-related to modulating inflammation, miR-16 was confirmed to negatively regulate the inflammation-related genes in LPS-induced NP cells, including COX-2, iNOS and PGE2 (51). Subsequently, target prediction found that TAB3 was directly regulated by miR-16, which was experimentally validated by a miRNA luciferase reporter assay. Besides, miR-16 attenuated the inflammation in LPS-mediated NP cells *via* inhibiting NF-κB and MAPK signal pathway. Likewise, MiR-223 was identified to share similar roles in LPS-treated NP cells through Irak1-mediated suppression of NF-κB (52). Apart from the above-mentioned, miR-15a-5p was found to aggravate the inflammation and apoptosis of NP cells by modulating NF-κB pathway (53). Hence, miRNAs can alleviate inflammation in NP cells by suppressing NF-κB. On the contrary, Dong et al. (54) demonstrated that miR-640 showed overt pro-inflammatory effects by enforcing activation of NF-κB, resulting in NP cells degeneration, conversely, inhibition of miR-640 displayed the opposite effects. Extracellular signal-regulated kinase (ERK) pathway has been evidenced to play key role in inhibiting inflammation (55). MiR-181a suppressed the expression of inflammatory factors through blocking ERK pathway in IDD mice (56), indicating that miR-181a affords protective effects in IDD.

Apart from mediated-apoptosis in IDD process, ERα is reported to a key player in modulating inflammation (57, 58). Specifically, the expression of miR-203-3p was positively correlated with the severity of IDD and negatively with Erα (58). Further evidence showed that ERα was the specific target of miR-203-3p and can be inhibited in LPS-stimulated NP cells, indicating that miR-203-3p was likely to aggravate intervertebral disc inflammation and degeneration through targeting ERα. These findings revealed that the same gene could exert different roles due to different miRNAs, implying the complexity and diversity of miRNA in regulation of IDD.

Additionally, other miRNAs were also demonstrated to link with inflammation in IDD progress. For example, miR-194-5p actively contributed to human IDD by targeting CUL4A and CUL4B and significantly decreased in inflammatory environment of IDD, indicating a negative regulation of miR-194-5p in the progression of IDD (59). In contrast, miR-125b-5p expression was found to be enforced in IL-1β-induced NP cells and human degenerating NP samples, which contributed to inflammation and NP cells apoptosis by monitoring TRIAP1 (60).

In summary, current studies demonstrate that miRNAs are key regulators and have important effects on the pathological cascades of IDD through intervening cells apoptosis, ECM metabolism and inflammation. In fact, the roles of miRNAs in the pathogenesis of IDD are not limited to the above-mentioned aspects. Latest studies indicate that miRNAs-mediated autophagy and ferroptosis are intimately linked with pathological progression of IDD. MiR-202-5p suppressed autophagy in degenerating NP cells through targeting ATG7 (61). Notably, Wu and colleagues (62) provided sufficient evidence that downregulation of miR-130b-3p promoted autophagy in NP cells and ameliorated IDD through ATG14/PRKAA1 *in vivo* and vitro. Ferroptosis, an iron-dependent type of programmed cell death, is associated with the pathogenesis of IDD. Overexpression of miR-10a-5p partially reversed IL-6-mediated ferroptosis in cartilage cells (63). In addition, latest evidence showed that inhibition of miR-874-3p positively modulated ferroptosis in NP cells by targeting activation transcription factor 3 (ATF3) (64). Undoubtedly, increasing studies revealed that miRNAs can mediate the initiation and progress of IDD (**Table 1**), indicating miRNAs-based therapy for IDD may be a promising strategy.

**Table 1:** The roles of miRNAs in the development of IDD.

MiRNAs	Expression	Target/Pathway	Function	Reference
miR-141	Up	SIRT1/NF-κB	NP cells apoptosis(+)	Ji et al. (19)
miR-96	Up	FRS2	NP cells apoptosis(+)	Yang et al. (20)
miR-4478	Up	MTH1	NP cells apoptosis(+)	Zhang et al. (21)
miR-328-5p	Up	WWP2	NP cells apoptosis(+)	Yan et al. (22)
miR-129-5p	Down	BMP2	NP cells apoptosis(-)	Yang et al. (23)
miR-623	Down	CXCL12	NP cells apoptosis/senescence(-)	Zhong et al. (24)
miR-155-3p	Down	KDM3A/HIF1α	NP cells apoptosis(-)	Zhou et al. (25)
miR-34a	Up	Bcl-2	CEP cells apoptosis(+)	Chen et al. (28)
miR-20a	Up	ANKH	CEP calcification(+)	Liu et al. (29)
miR-221	Up	ERα	CEP cells apoptosis(+)	Sheng et al. (30)
miR-142-3p	Down	HMGB1	CEP cells apoptosis(-)	Wang et al. (31)
miR-106a-5p	Up	ATG7	AF cells apoptosis(+)	Hai et al. (32)
miR-127-5p	Down	MMP-13	ECM anabolism(+)	Hua et al. (34)
miR-210	Up	ATG7/MMP-13/MMP-3	ECM catabolism(+)	Wang et al. (35)
miR-21	Up	PTEN/Akt/mTOR/MMP-9/MMP-3	ECM catabolism(+)	Wang et al. (36)
miR-132	Up	GDF5	ECM catabolism(+)	Liu et al. (39)
miR-665	Up	GDF5	ECM catabolism(+)	Tan et al. (40)
miR-30d	Up	SOX9	NP cells apoptosis/ECM catabolism(+)	Lv et al. (42)
miR-499a-5p	Down	SOX4	NP cells apoptosis/ECM catabolism(-)	Sun et al. (43)
miR-154	Up	FGF14	ECM catabolism(+)	Wang et al. (44)
miR-145	Down	ADAM17	NP cells apoptosis(-)/ECM anabolism(+)	Zhou et al. (45)
miR-1260b	Down	TCF7L2	ECM anabolism(+)	Chen et al. (46)
miR-16	Down	TAB3	Anti-inflammation	Du et al. (51)
miR-223	Down	Irak1	Anti-inflammation	Wang et al. (52)
miR-15a-5p	Up	SOX9/NF-κB	Pro-inflammation/NP cells apoptosis(+)	Zhang et al. (53)
miR-640	Up	NF-κB	Pro-inflammation	Dong et al. (54)
miR-181a	Down	ERK	Anti-inflammation	Sun et al. (56)
miR-203-3p	Up	ERα	Pro-inflammation	Cai et al. (58)
miR-194-5p	Down	CUL4A/CUL4B	Anti-inflammation	Chen et al. (59)
miR-125b-5p	Up	TRIAP1	Pro-inflammation/NP cells apoptosis(+)	Jie et al. (60)
miR-202-5p	Up	ATG7	NP cells autophagy(-)	Chen et al. (61)
miR-130b-3p	Up	ATG14/PRKAA1	NP cells autophagy(-)	Wu et al. (62)
miR-10a-5p	Down	IL-6R	Cartilage cells ferroptosis(-)	Bin et al. (63)
miR-874-3p	Down	ATF3	NP cells ferroptosis(-)	Li et al. (64)

(+), promotion; (-), inhibition.

## The roles of lncRNAs in the development of IDD

LncRNAs are a class of long noncoding RNAs whose transcript length exceeds 200 nucleotides in length. In general, lncRNAs are lack of capacity to code proteins, but share excellent regulation of gene expression through genetic, transcriptional and post-transcriptional modifications (65). Recently, emerging studies indicated that lncRNAs were associated with musculoskeletal degeneration diseases, such as osteoarthritis and IDD (4, 66). LncRNAs have exerted critical effects on the IDD by regulating cellular phenotype (cells proliferation, apoptosis, autophagy and ECM metabolism) through directly targeting hub gene expression and miRNAs.

It is well known that lncRNAs participate in the pathological changes of degenerative diseases by sponging specific miRNAs. LncRNA PART1 was verified to be significantly increased in NP cells isolated from IDD patients, indicating that PART1 probably impacted the degeneration of NP cells (67). Then, the expression of genes responsible for cells apoptosis, proliferation and ECM metabolism were evaluated. Results showed that lncRNA PART1 promoted NP cells apoptosis and ECM degradation, whereas cells proliferation and ECM synthesis were suppressed. Mechanistically, lncRNA PART1 competitively sponged miR-93, causing the degradation of NP cells by MMP2. Based on these findings, it can be inferred that lncRNAs play key role in IDD, albeit lack of *in vivo* evidence to support the potential role of PART1 for IDD. Zhong and team (68) found that lncRNA ADIRF-AS1/miR-214-3p/SERPINA1 pathway was negatively correlated with the severity of IDD, suggesting the protective role of ADIRF-AS1. Specifically, upregulation of ADIRF-AS1 enhanced NP cells viability and suppressed cellular senescence and apoptosis. Strikingly, latest study reported by Yu et al. (69) uncovered that lncRNA GAS5 was a principal contributor to NP cells apoptosis and catabolism of ECM through miR-17-3p/Ang-2 axis, eliciting the occurrence and progress of IDD. In addition, both inhibition of GAS5 and upregulation of miR-17-3p ameliorated IVDD in mice models. Collectively, *in vitro* and vivo studies afforded abundant supports that the lncRNA GAS5 might participate in IDD progress by miR-17-3p/Ang-2-mediated NP cells apoptosis and ECM degradation. Besides, inflammation and oxidative stress destroyed the homeostasis of IVD, enhancing the development of IDD. LncRNA MT1DP was found to mitigate anti-oxidation through miR-365/NRF-2 signal pathway, leading to NP cells apoptosis and IDD (70). Notably, LncRNA FAM83H-AS1 alleviated inflammatory response and promoted NP cells proliferation through miR-22-3p, preventing further deterioration of IDD in rat models caused by advanced glycation end products (71).

LncRNAs play important roles in regulating IDD through direct modulation of hub gene or signal pathway, ultimately triggering downstream cascades. Within the event, nuclear factor E2-related factor 2 (Nrf2) was reported to facilitate protecting NP cells from oxidative injury and preventing IDD deterioration (72, 73). Kang and colleagues (74) evidenced that upregulation of lncRNA ANPODRT attenuated oxidative stress and reduced tert-butyl hydroperoxide-stimulated apoptosis in NP cells, which could be attributed to the activation of Nrf2. As previously mentioned, miRNAs-mediated autophagy influenced the progress of IDD. As a consequence, lncRNAs may affect the process of IDD through autophagic pathway. HOTAIR, a novel lncRNA, was found to associate with autophagy and highly expressed in human NP tissue suffering from IDD (75). Furthermore, upregulation of HOTAIR potentiated autophagy *via* AMPK/mTOR/ULK1 signaling pathway in human NP cells, leading to NP cells apoptosis, senescence, and ECM degradation. More importantly, blocking HOTAIR attenuated the adverse effects in IDD rats. Interestingly, lncRNA HOTAIR could activate Wnt/β-catenin pathway and exert similar functions except for modulating AMPK/mTOR/ULK1 (76), implying lncRNA may act as key regulators of multiple downstream pathways.

At the present, an increasing number of lncRNAs have been found to involve in the pathological process of IDD, such as lncRNA SNHG6/miR-101-3p (77), lncRNA MIR155HG/miR-223-3p (78) and lncRNA H19/miR-139-3p/CXCR4/NF-κB (79) axes. In summary, these important roles of lncRNAs in IDD have been comprehensively verified (**Table 2**). Helpfully, these findings further elucidate the underlying mechanisms of IDD and shed light on lncRNAs as potential therapeutic target for treatment of IDD.

**Table 2:** The roles of lncRNAs in the development of IDD.

LncRNAs	Expression	Target/Pathway	Function	Reference
lncRNA PART1	Up	miR-93/MMP2	NP cells apoptosis/ECM catabolism(+)	Gao et al. (67)
lncRNA ADIRF-AS1	Down	miR-214-3p/SERPINA1	NP cells apoptosis/senescence(-)	Zhong et al. (68)
lncRNA GAS5	Up	miR-17-3p/Ang-2	NP cells apoptosis/ECM degradation(+)	Yu et al. (69)
lncRNA MT1DP	Up	miR-365/NRF-2	NP cells apoptosis(+)	Liao et al. (70)
lncRNA FAM83H-AS1	Down	miR-22-3p	Anti-inflammation/NP cells growth(+)	Jiang et al. (71)
lncRNA ANPODRT	Down	Nrf2	Anti-inflammation	Kang et al. (74)
lncRNA HOTAIR	Up	AMPK/mTOR/ULK1	NP cells apoptosis/senescence(+)	Zhan et al. (75)
lncRNA HOTAIR	Up	Wnt/β-catenin	NP cells apoptosis/ECM degradation(+)	Zhan et al. (76)
lncRNA SNHG6	Up	miR-101-3p	NP cells apoptosis(+)	Gao et al. (77)
lncRNA MIR155HG	Up	miR-223-3p	NP cells pyroptosis(+)	Yang et al. (78)
lncRNA H19	Up	miR-139-3p/CXCR4/NF-κB	NP cells apoptosis(+)	Sun et al. (79)

(+), promotion; (-), inhibition.

## The roles of circRNAs in the development of IDD

CircRNAs, another particular type of ncRNAs with the covalently closed loops, have attracted substantial attentions because of their excellent biological properties. Currently, there are a series of circRNAs have been evidenced to be pertinent to the underlying mechanisms of IDD (15, 80). As naturally formed endogenous non−coding RNAs, circRNAs serve as a competing endogenous RNAs and modulate the pathological process of IDD, mainly involving in cellular apoptosis, ECM metabolism and inflammation (80).

In the light of pathological mechanism of IDD, NP cells apoptosis is primary factor accelerating the pathological progression of IDD. Cheng and colleagues (81) observed that circVMA21 could depress the expression of apoptotic and catabolic genes, and enhance the collagen II and aggrecan expression in NP cells treated by inflammatory cytokines through miR-200c/XIAP pathway. *In vivo* injection of circVMA21 ameliorated the degeneration of NP tissues in rat model, suggesting the protective role of circVMA21/miR-200c/XIAP against IDD. Subsequently, Guo et al. (82) also verified that circGRB10 provided the beneficial effects in preventing IDD by reducing NP cell apoptosis through miR-328-5p/ERBB2 axis. Similarly, other studies demonstrated that cicrRNAs also afforded protection against IDD development, mainly including circRNA-CIDN/miR-34a-5p/SIRT1 (83), circGLCE/miR-587/STAP1 (84) and circARL15/miR-431-5p/DISC1 (85) pathway. On the contrary, recent studies showed that circRNAs facilitated apoptosis of NP cells. For instance, circ_001653 was significantly upregulated in degenerated NP tissues, which promoted the NP cells apoptosis and ECM degradation by miR-486-3p/CEMIP axis (86). In addition, a series of evidence indicated that circITCH induced apoptosis and mediated IDD through miR-17-5p/SOX4 axis (87).

As is known to all, the imbalance of ECM metabolism is detrimental for IDD. Considering that miRNAs play important role in modulating anabolism and catabolism of ECM, circRNAs also have been reported to associate with ECM metabolism through sponging miRNAs. Upregulation of circ-4099 promoted the ECM (collagen II and aggrecan) synthesis and restricted expression of pro-inflammatory factors (IL-1β, TNF-α, and PGE2) through targeting miR-616-5p/SOX9 (88). Likewise, circSEMA4B could alleviate ECM catabolism in IL-1β-induced NP cells *via* miR-431/GSK-3β/SFRP1 axis (89). Accordingly, these results support that circRNAs can prevent IDD through facilitating the anabolism of ECM. Nevertheless, circRNAs also share unfavorable roles in ECM metabolism and can induce ECM degradation. For instance, growing evidence showed that circRNA_104670 enhanced the expression of MMP-2 known to be associated with ECM catabolism, through miRNA-17-3p/MMP2 pathway, potentiating ECM degradation (90). Furthermore, circRNA_104670 and miRNA-17-3p was found to have excellent diagnostic significance for IDD based on the outcome of the receiver-operating characteristic curve.

Recently, accumulating studies found that circRNAs play vital roles in modulating inflammation response. It is well known that NF-κB is a classical inflammation-related pathway. Guo et al. (91) evidenced that circ-FAM169A mediated inflammatory cytokines (IL-1β and TNF-α) expression through BTRC/NF-κB axis, which induced NP cells apoptosis and ECM degradation. Based on above, there is no doubt that circRNAs play a crucial role in the regulation of miRNAs. Notwithstanding, whether multiple circRNAs can interplay and impact the biological process remain unclear. Strikingly, latest publication showed that circ_0040039 and circ_0004354 competitively regulated miR-345-3p/FAF1/TP73 pathway in IDD, initiating inflammation, ECM catabolism and pro-inflammation in NP cells (92). In detail, circ_0004354 showed stronger capacity to bind miR-345-3p when inflammatory cytokines reached lower level at the early phase of IDD. Subsequently, circ_0004354 was suppressed through negative feedback due to increase of inflammatory cytokines. Meanwhile, increased inflammatory cytokines induced circ_0040039 expression, which in turn inhibited the binding of circ_0004354 with miR-345-3p and augmented circ_0040039 ability to target miR-345-3p. Collectively, circ_0004354 and circ_0040039 showed different capacity to bind miR-345-3p relying on different concentration of inflammatory cytokines, eventually triggering inflammatory cascades and accelerating IDD progression.

Taken together, circRNAs display excellent ability to modulate the development of IDD through cells apoptosis, ECM metabolism and inflammation (**Table 3**). In addition, circRNAs also take important roles in affecting cellular senescence. For instance, latest research published by Wang et al. (93) reported that circ_7042 could prevent the IDD progression through inhibiting NP cells apoptosis, senescence and ECM degradation by absorption of miR-369-3p/BMP2/PI3K/Akt axis. Apart from sponging miRNAs, the interaction of different circRNAs may have impacts on the downstream pathway, which urgently needs more evidence to further verify.

**Table 3:** The roles of circRNAs in the development of IDD.

CircRNAs	Expression	Target/Pathway	Function	Reference
circVMA21	Down	miR-200c/XIAP	NP cells apoptosis(-)/ECM anabolism(+)	Cheng et al. (81)
circGRB10	Down	miR-328-5p/ERBB2	NP cells apoptosis(-)	Guo et al. (82)
circRNA-CIDN	Down	miR-34a-5p/SIRT1	NP cells apoptosis/ECM catabolism(-)	Xiang et al. (83)
circGLCE	Down	miR-587/STAP1	NP cells apoptosis/ECM catabolism(-)	Chen et al. (84)
circARL15	Down	miR-431-5p/DISC1	NP cells apoptosis(-)	Wang et al. (85)
circ_001653	Up	miR-486-3p/CEMIP	NP cells apoptosis/ECM catabolism(+)	Cui et al. (86)
circITCH	Up	miR-17-5p/SOX4	NP cells apoptosis/ECM catabolism(+)	Zhang et al. (87)
circ-4099	Down	miR-616-5p/SOX9	ECM anabolism(+)	Wang et al. (88)
circSEMA4B	Down	miR-431/GSK-3β/SFRP1	ECM anabolism(+)	Wang et al. (89)
circRNA_104670	Up	miRNA-17-3p/MMP2	ECM catabolism(+)	Song et al. (90)
circ-FAM169A	Up	miR-583/BTRC/NF-κB	ECM catabolism(+)	Guo et al. (91)
circ_0040039/circ_0004354	Up	miR-345-3p/FAF1/TP73	Inflammation/ECM catabolism(+)	Li et al. (92)
circ_7042	Down	miR-369-3p/BMP2/PI3K/Akt	NP cells apoptosis/senescence/ECM degradation	Wang et al. (93)

(+), promotion; (-), inhibition.

## Interactions of miRNAs, lncRNAs, and circRNAs

**Figure 2:**
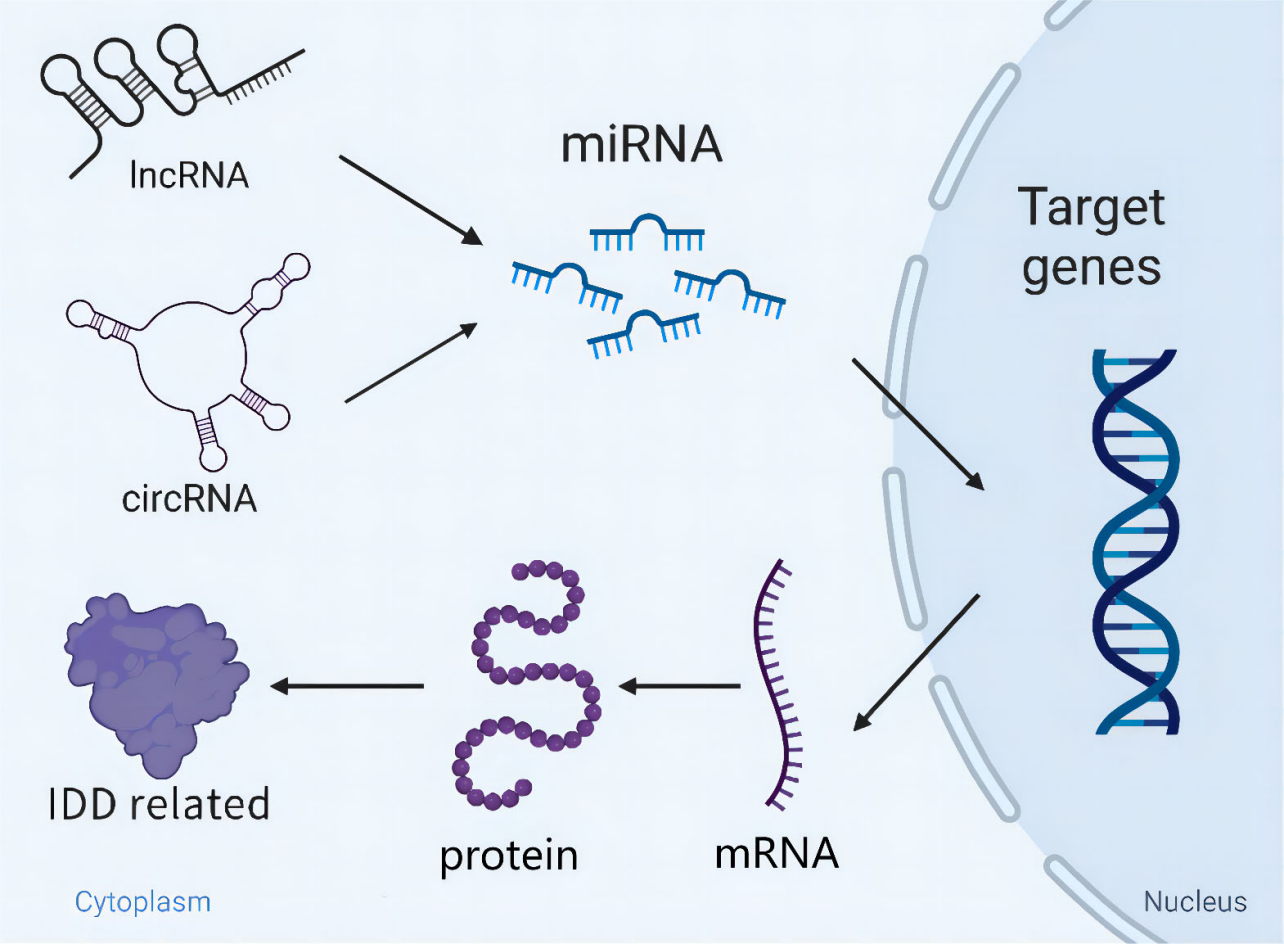
Schematic diagram of the cross-talk of miRNAs/lncRNAs/circRNAs orchestrating IDD development. LncRNAs/circRNAs directly sponge miRNAs and initiate a series of gene expression relating to IDD. Besides, miRNAs are able to modulate IDD independently through targeting key genes.

In terms of current findings, dysregulation of ncRNAs mediates the onset and progression of IDD. More importantly, the exertion of functions regarding ncRNAs appears to be not completely independent of modulating the pathological process of IDD. LncRNAs and circRNAs, as particular ncRNAs, can directly sponge miRNAs and initiate a series of gene expression to regulate cells apoptosis, ECM metabolism and inflammation. Furthermore, there is a competitive relationship to bind miRNAs among multiple circRNAs. Collectively, the cross-talk of miRNAs/lncRNAs/circRNAs orchestrates IDD development (**Figure 2**), which is similar to the network and can modulate each other.

## Therapeutic strategies for IDD based on ncRNAs

**Figure 3:**
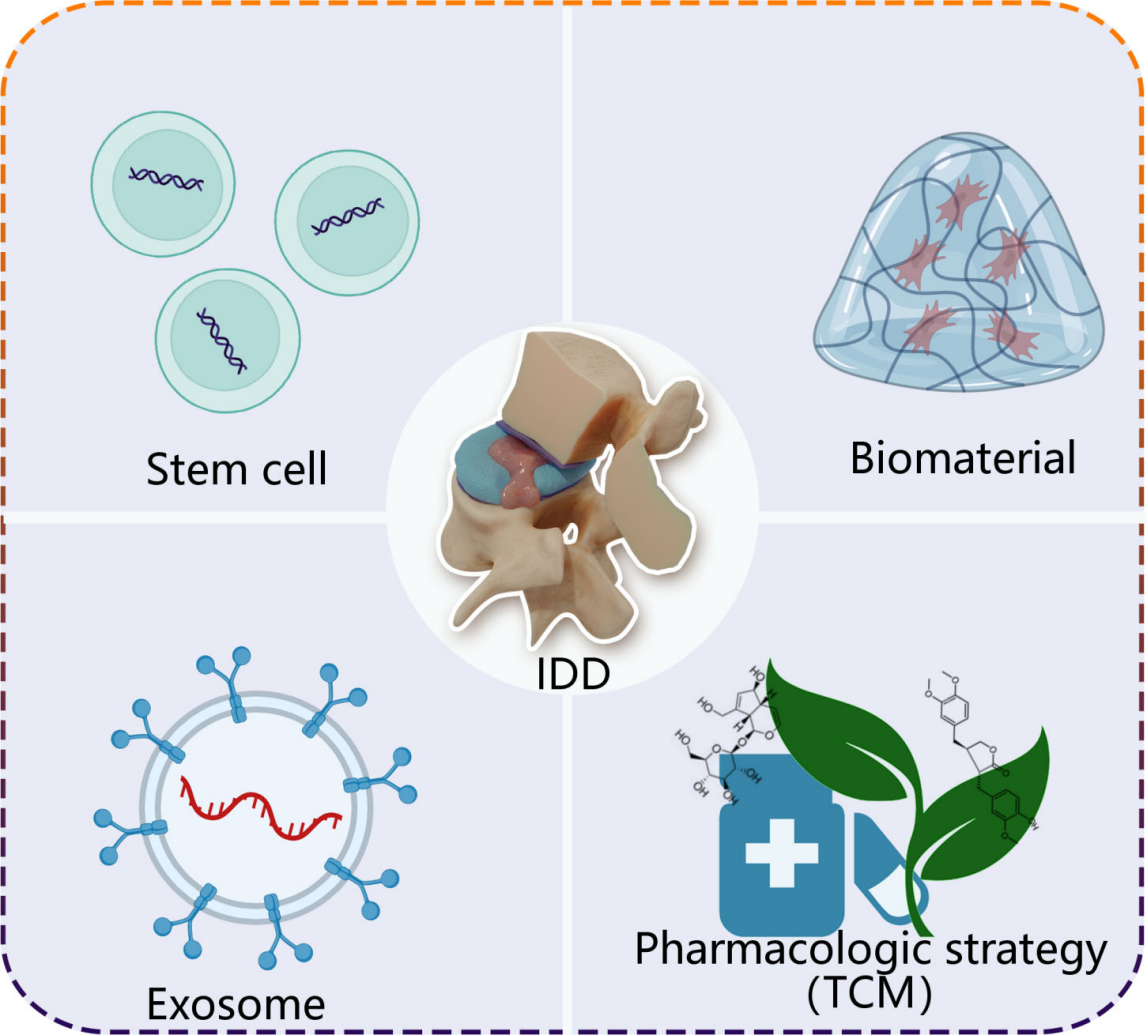
A simplified scheme of ncRNA-based therapeutics for treatment of IDD. Stem cell, exosome, biomaterial and pharmacologic strategy, as carries, deliver loading endogenous and exogenous ncRNAs to degenerative intervertebral disc tissue. TCM, traditional Chinese medicine.

On the basis of understanding functions of ncRNAs, emerging evidence demonstrate that multiple strategies show excellent efficacy on treatment IDD through ncRNAs, mainly including stem cell therapy, exosomes, biomaterials and pharmacologic strategy (**Figure 3**).

### Stem cell

With more profound studies on stem cells, stem cell therapy is considered as a promising approach to intervene IDD owing to their capacity to release ncRNAs (94, 95). Shi and colleagues (96) found that bone marrow mesenchymal stem cells (BMSCs) enhanced the expression of autophagy-related genes and suppressed apoptosis-related genes in OGD NP cells through secreting miR-155, reducing NP cells apoptosis. Although they indicated that miRNA-derived from BMSCs probably afforded protective effects on NP cells *in vitro*, there is currently no *in vivo* evidence. Therefore, whether stem cells can improve IDD by ncRNAs needs further study. Intriguingly, a recent study demonstrated that BMSC-derived extracellular vesicles (BMSC-EVs) supported survival of NP cells and reduced ECM catabolism through circ_0050205/miR-665/GPX4 pathway in IDD mice (97). Hence, stem cell therapy may also be an effective strategy for treatment of IDD through ncRNAs.

### Exosome

Exosome, a kind of extracellular vesicles secreted by the majority of cell types, contains numerous bioactive components involved in intercellular communication. Accumulating studies have paid attention to the role of exosomal ncRNAs in treatment of IDD (98, 99). For instance, Cheng and colleagues (100) demonstrated that MSC-derived exosomes (MSC-exosomes) could be taken by NP cells and exerted cytoprotective effects against NP cells apoptosis. This was likely to be mainly ascribed to activation of miR-21/PI3K/Akt signal pathway. In rat IDD model, the injection of MSC-exosomes reduced NP cells apoptosis and delayed IDD deterioration, showing the therapeutic potential of MSC exosomes in IDD. Recently, Chen et al. (101) also extracted exosomes from cartilage endplate stem cells (CESC-exosomes) and found that CESC-exosomes can suppress NP cells apoptosis and ECM degradation through delivery of miR-125-5p. Although exosomal ncRNAs show greatly therapeutic potential in treatment of IDD, obtaining highly purified exosomes in large quantities still requires further investigation. Hence, further study needs to be conducted to bail out current dilemma for clinical application.

### Biomaterial

Biomaterial-based strategies have attracted increasing attention in the field of disc pro-regeneration (102, 103). Existing studies indicate that biomaterials have shown remarkable potential to arrest IDD owing to their unique biological properties including excellent biocompatibility and mechanical properties (103). Hydrogel, a particular biomaterial, is similar to the natural extracellular matrix and has been widely used to clinical trials. Feng and colleagues (104) designed a kind of polyplex micelle-encapsulated hydrogel that could encapsulate miR-29a and prevent miR-29a from spillage and degradation *in vivo*. In rabbit IDD models, the injection of miR-29a/polyplex reversed IDD through the suppression of MMP-2/β-catenin signal pathway. Lipid nanocapsules (LNC), as a particular carrier, can load and release certain miRNAs into cells. Regarding the point, miR-155-loaded LNC (miR-155 LNC) has been devised to evaluate the potential for treatment of IDD *in vitro* and *in vivo* (105). Intriguingly, their results showed that miR-155 LNC could be internalized in NP cells and maintain bioactivity. Moreover, *in vivo* experiments, injection of miR-155 LNC was proven to be safe and feasible. Unfortunately, they didn’t further investigate the specific effects of miR-155 LNC on IDD progress. Collectively, biomaterials show great potential for treatment of IDD through delivery of ncRNAs.

### Pharmacologic

At the present, pharmacological intervention is also considered as adjuvant therapy alternatives for IDD, such as traditional Chinese medicine (TCM) and natural products (106, 107). Notably, Yang et al. (108) found that aucubin, an active ingredient of eucommia ulmoides, could prevent the ECM degradation in human NP cells treated by IL‐1β or TNF‐α. Concomitantly, further experiments verified that miR-140/CREB1 signal pathway participated in aucubin-mediated protection against IDD (108). In addition, latest study demonstrated that arctigenin shared multiple roles in anti-IDD through up-regulating miR-483-3p, including reduction of NP cells apoptosis, ECM catabolism and inflammation-related genes expression (109). Consequently, these vitro findings afford a novel pharmacological strategy based on ncRNAs for treatment IDD. Although ncRNAs can be envisioned as pharmacological targets for the management of IDD, whether pharmacologic strategy-linked with ncRNAs exerts positive effects *in vivo* due to the complicated internal environment. Therefore, future study should focus on the *in vivo* effects and screen the determined ncRNAs for potential treatment of IDD.

Up to now, ncRNA-based therapeutics have made enormous progress, but present studies focused only on NP cells or rodents with IDD models. In fact, the biomechanical properties of intervertebral disc are obviously different between human and rodents. Therefore, it is essential to investigate the efficacy of ncRNA-based therapy in the IDD models conforming to the human biomechanical properties.

## Prospects

Intervertebral disc degeneration is a main contributor to chronic low back pain. With regard to the pathological features, many factors involved in the progression of IDD, mainly focused on cells apoptosis, ECM metabolism and inflammation (9, 11). Increasing evidence have indicated that ncRNAs involve in the initiation and development of IDD, which hints the important potential of ncRNAs for IDD of treatment (110). In present review, we summarized the functions of ncRNAs in IDD and found that ncRNAs acted as pivotal regulators in the pathological process of IDD. On one hand, ncRNAs play positive roles in delaying or reversing IDD progression through inhibiting cells apoptosis, ECM catabolism and inflammatory cascades. On the other hand, ncRNAs also cause IDD deterioration by promoting cells apoptosis, ECM degradation and pro-inflammatory cytokines secretion. In addition, ncRNAs have exerted effects on IDD by regulating autophagy and ferroptosis. In fact, the ncRNAs to IDD is a double-edge sword, which depends on the downstream hub gene or signal pathway. Critically, multiple ncRNAs can regulate the same target genes or the different targets can be modulated by the same ncRNAs, like network, which jointly participates in IDD. Based on the existing evidence, ncRNAs have been attempted to manage the degenerative processes and shown positive efficacy though delivery of ncRNAs.

To our best knowledge, it is requisite for clinical application to develop drug targeting ncRNAs and conduct large-scale studies. Current studies demonstrate that a variety of ncRNAs are involved in the process of IDD (15, 80), but the decisive ncRNAs in IDD remain unclear. Furthermore, most studies still focused on the NP cells or IDD models. Therefore, ncRNAs-based therapy is still in the preclinical stage. Apart from clinical treatment, whether ncRNAs can be considered as clinical biomarkers for diagnosis is not fully elucidated, diagnosis of IDD at early stage is still challenging. In the future, research should pay more attention to the following aspects: i). the roles of ncRNAs should be explored in depth to better understand the underlying mechanisms of IDD; ii). screening of the ncRNAs with diagnostic value; iii). design of the IDD models that are similar to human biomechanical properties.

In conclusion, the present studies have demonstrated that ncRNAs are hub regulators mediating the onset and progression of IDD. In this review, we have systematically reviewed recent advances in related fields and summarized the role of ncRNAs in IDD. Further, we have discussed the ncRNAs-based strategy for treatment of IDD, which sheds light on the preface of switching theoretical strategy toward actually clinical application. Lastly, considering the current research advance in ncRNAs for treatment of IDD, we have analyzed the issues that need to be paid more attention in future research, producing meaningful ideas for next studies. Based on the aforementioned, ncRNAs, as novel therapeutic targets for IDD, may possess an excellent prospect. Comprehensive understanding the function of ncRNAs in IDD is critical for exploring biological therapies for treatment of IDD.

## Author contributions

HY and DH conceived of the review and supervised the project. CJ, ZC and XW wrote the manuscript and drew figures. YZ and XG contributed to literature review and editing. ZX contributed to compiled table. All authors contributed to the article and approved the submitted version.
